# Effects of Persistent Binge Drinking on Brain Structure in Emerging Adults: A Longitudinal Study

**DOI:** 10.3389/fpsyt.2022.935043

**Published:** 2022-06-23

**Authors:** Jose Manuel Pérez-García, Fernando Cadaveira, Erick J. Canales-Rodríguez, Samuel Suárez-Suárez, Socorro Rodríguez Holguín, Montserrat Corral, Javier Blanco-Ramos, Sonia Doallo

**Affiliations:** ^1^Department of Clinical Psychology and Psychobiology, Universidade de Santiago de Compostela (USC), Santiago de Compostela, Spain; ^2^Signal Processing Laboratory 5 (LTS5), École Polytechnique Fédérale de Lausanne (EPFL), Lausanne, Switzerland; ^3^FIDMAG Germanes Hospitalàries Research Foundation, Sant Boi de Llobregat, Spain; ^4^Centro de Investigación Biomédica en Red de Salud Mental (CIBERSAM), Madrid, Spain

**Keywords:** binge drinking, brain structure, longitudinal, sex differences, surface-based morphometry, emerging adulthood

## Abstract

Previous cross-sectional research has largely associated binge drinking (BD) with changes in volume and thickness during adolescence and early adulthood. Nevertheless, the long-term alcohol-related effects on gray matter features in youths who had maintained a BD pattern over time have not yet been sufficiently explored. The present study aimed to assess group differences both cross-sectionally and longitudinally [using symmetric percent change (SPC)] on several structural measures (i.e., thickness, surface area, volume). For this purpose, magnetic resonance imaging was recorded twice within a 2-year interval; at baseline (18–19 years) and a follow-up (20–21 years). The sample included 44 university students who were classified as 16 stable binge drinkers (8 females) and 28 stable controls (13 females). Whole-brain analysis showed larger insular surface area in binge drinkers relative to controls at follow-up (cluster-wise *p* = 0.045). On the other hand, region of interest (ROI) analyses on thickness also revealed a group by sex interaction at follow-up (*p* = 0.005), indicating that BD males had smaller right rostral middle frontal gyrus thickness than both control males (*p* = 0.011) and BD females (*p* = 0.029). Similarly, ROI-based analysis on longitudinal data showed a group by sex interaction in the right nucleus accumbens (*p* = 0.009) which revealed a decreased volume across time in BD males than in control males (*p* = 0.007). Overall, continued BD pattern during emerging adulthood appears to lead to gray matter abnormalities in regions intimately involved in reward processing, emotional regulation and executive functions. Notably, some anomalies varied significantly depending on sex, suggesting a sex-specific impact of BD on typical neurodevelopment processes.

## Introduction

Alcohol is the most widely available and commonly used drug during adolescence and youth, as informed by epidemiological surveys (1, 2). Among the various alcohol consumption patterns, binge drinking (BD) is the most prevalent among young adults. It has been associated with several neuropsychological, structural, and functional anomalies (3–5), as well as with an increased risk of developing alcohol use disorder (6–8). This type of drinking is characterized by the intake of large amounts of alcohol in a brief time followed by intervals of abstinence, and it is generally defined as the consumption of 5 or more drinks (4 or more for females) on one occasion within a 2-h time period (which leads to a blood alcohol concentration of at least 0.08 g/dL) (9). Notably, peak BD rates are reached between ages 18 and 25 (2), a life-changing period in which brain maturation is still under development and may be particularly vulnerable to the neurotoxic effects of alcohol (10–13).

When interpreting the results of studies that have assessed how alcohol intake affects the adolescent brain, we must also consider the differences and overlap between BD and other types of consumption. Although the definition of BD has long been debated, in the present work, we consider the combination of rapid and intermittent intoxication episodes with periods of abstinence as a key feature to characterize this pattern, as proposed by Maurage et al. (14). On this basis, the confusion around BD pattern conceptualization is reduced, allowing us to distinguish the effects of BD from those of other drinking patterns, for instance heavy drinking, which refers to consumption of alcohol more frequently (14, 15).

Neuroanatomical differences in both binge drinkers (BDs) and young people with heavy drinking relative to controls have been extensively reported by magnetic resonance imaging (MRI) studies, suggesting that the two drinking behaviors are associated with distinct gray matter changes (5, 16–18). As our interest is focused on exploring specific effects of BD, we set for our sample strict inclusion and exclusion criteria to better isolate the effects of BD on the developing brain.

Previous cross-sectional studies that have used specific BD criteria to characterize the impact of alcohol consumption during adolescence and young adulthood have consistently shown that this pattern can lead to common gray matter abnormalities (in volume and/or thickness) in prefrontal regions (i.e., middle frontal gyrus and anterior cingulate) and subcortical limbic areas (i.e., ventral striatum/accumbens) intimately involved in the control and regulation of impulsive or risky behaviors, as well as in processing rewarding stimuli (19–24). Likewise, sex differences in the effect of BD on brain structure, including but not limited to prefrontal regions, have also been identified in other studies which reported both reduced volume and lower thickness in frontal, striatal, middle temporal, and parietal regions in male BDs than their control peers, while female BDs showed the opposite pattern (25, 26). It is worth noting that the significance of the directionality of the anomalies is not clear yet. It has been hypothesized that either the increase or decrease in gray matter features (observed in BDs relative to controls in these studies) may represent an alteration in typical neurodevelopment, probably caused by the neurotoxic effects of alcohol (27, 28).

Longitudinal investigations that have attempted to shed light on the consequences of excessive alcohol consumption on the brain have revealed altered developmental trajectories in young heavy drinkers relative to controls (27, 29–34). Specifically, studies that have examined neural structure both before and after the onset of heavy drinking reported recurrent accelerated gray matter declines, particularly in frontal (27, 29, 33, 34) and temporal regions (32, 33). On the other hand, in line with some of the longitudinal studies stated above, two works exploring the effects of continuous heavy drinking among emerging adults, who were heavy drinkers at baseline, also informed of gray matter loss in frontal and temporal (30) as well as subcortical regions (31). Remarkably, similar frontal findings were revealed in a study that performed a single MRI scan after a 10-year history of heavy alcohol consumption (35). However, most of these studies recruited young people with a history of heavy drinking. Therefore, a study whose participants have a specific BD pattern as the main consumption behavior is necessary.

Taking into account these considerations, we aimed to assess whether the possible anomalies related to a BD pattern are maintained or increased over a 2-year follow-up period. Thus, we compared brain trajectories of university students who maintained a stable BD pattern vs. their control peers in three gray matter cortical features, including thickness, volume, and surface area, as well as subcortical volumes, through surface-based morphometry (SBM) method. Interestingly, recent studies have suggested that thickness and surface area are heritable but genetically and phenotypically independent (36, 37) and regulated by different processes (37, 38). To carry out the longitudinal analysis, we applied a robust measure [i.e., symmetric percent change (SPC)] that has not been used before in BD research to explore cortical and subcortical development changes over time. In addition, another important goal of this study was to examine potential sex-related differences on the effects of the maintenance of the BD pattern.

Accordingly, we tested three hypotheses. First, based on the aforementioned cross-sectional findings, we predicted that young BDs would exhibit alterations in gray matter morphology indices compared to controls at baseline, specifically in brain regions that showed anomalies in at least two independent studies, such as anterior cingulate, middle frontal gyrus, and ventral striatum/accumbens. Second, we expected that these potential abnormalities would be more pronounced at follow-up and that other initially unobserved anomalies may emerge due to maintaining the pattern of consumption. Third, we hypothesized that differences in SPC rate would be found between BDs and controls. No specific directional hypotheses were advanced regarding the examined structural metrics, given the inconsistent findings from previous research.

## Materials and Methods

### Participants

The final sample comprised 44 participants who were enrolled in a longitudinal neuroimaging study within the framework of a broader research project on consequences of BD among university students (39–41). All subjects completed MRI assessments at two different times; at baseline and a follow-up when they were aged 18–19 and 20–21 years, respectively.

**FIGURE 1:**
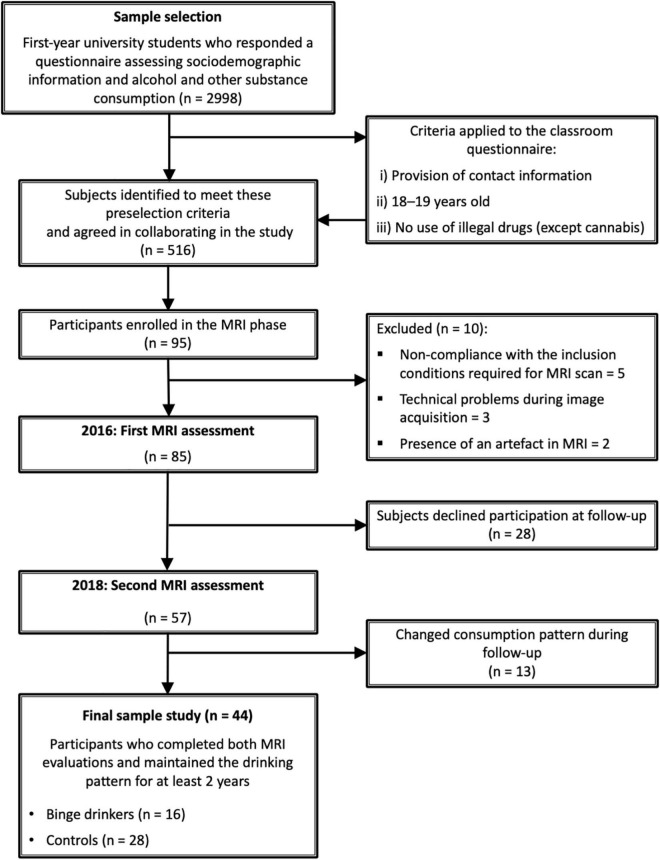
Timeline with the phases of the study.

Initially, 2,998 first-year students from the University of Santiago de Compostela (USC, Spain) were selected based on their responses to a classroom questionnaire assessing sociodemographic information and alcohol and other substance consumption (the study’s timeline is shown in Figure 1). The questionnaire included the Galician adapted version of the Alcohol Use Disorders Identification Test (AUDIT) (42, 43), the short version of the Nicotine Dependence Syndrome Scale (NDSS-S) (44, 45) and the Cannabis Abuse Screening Test (CAST) (46, 47). To distinguish the most suitable subjects from the initial 2,998 questionnaires, the following pre-selection criteria were applied to the classroom questionnaire: (i) provision of contact information (telephone number and e-mail); (ii) 18–19 years old; and (iii) no illicit drug use except cannabis. From this sample, 516 subjects who met these criteria agreed to participate in the study. These participants then completed a semi-structured interview and a set of questionnaires including medical history, the Alcohol Timeline Follow-back calendar (TFLB) over the past 180 days, the Cannabis TLFB over the past 90 days (48), and the Spanish version of Symptom Checklist-90-Revised (SCL-90-R) (49). Those interviewees who met the inclusion/exclusion criteria (see Table 1) were considered for enrolment in the MRI phase. All participants gave written consent and received monetary compensation for their collaboration. The study was approved by the Bioethics Committee of the USC.

**TABLE 1:** Exclusionary criteria established in the study.

Exclusion criteria
• Medical conditions affecting the normal cognitive functioning (hypothyroidism, diabetes, etc.) • History of neurological disorders or history of brain injury with loss of consciousness for longer than 20 min • History of diagnosed psychopathological disorders (axis I and II, according to DSM-IV-TR criteria) • SCL-90-R score > 90th percentile on Global Severity Index (GSI) or at least two symptomatic dimensions • Family history of major psychopathological disorders in first-degree relatives (clinically diagnosed by a professional) • Family history of first-degree alcoholism or substance abuse • Non-corrected sensory deficits and MRI contraindications • AUDIT scores^a^ > 20 at the start of the study • Regular consumption of substances with psychoactive effects (psycholeptics) • Use of illegal drugs (except occasional consumption of cannabis)^b^

***^a^**AUDIT, Alcohol Use Disorders Identification Test.*

***^b^**Subjects who consumed > 12 units over the last 90 days, or who regularly consumed cannabis (1 or more units per week), were not included.*

Subjects were classified as BDs if they reported one BD episode at least once a month over the last 6 months, or as controls if they did not meet the alcohol consumption threshold to be considered BDs. A BD episode was defined as the consumption of ≥ 50 g (females) or ≥ 70 g (males) of alcohol in one occasion, raising blood alcohol concentration above 0.08 g/dL (i.e., a measure that corresponds to the 4/5 standard drinks criteria specified in the NIAAA’s definition of BD) (9). As the objective in this study was to compare longitudinal trajectories of consumption, only participants who met these criteria fulfilled in both time evaluations were selected. Hence, the final sample included 44 participants, with 16 continuous BDs (8 females) and 28 continuous controls (13 females). The sociodemographic and drinking characteristics of each group are summarized in Table 2.

**TABLE 2:** Demographic and alcohol use characteristics of the control and BD groups (mean [95% CI]).

	Baseline		Follow-up	
	Controls	BDs	Controls	BDs
*n* (females)	28 (13)	16 (8)	28 (13)	16 (8)
Age	18.55 [18.44–18.66]	18.56 [18.40–18.73]	20.57 [20.44–20.70]	20.51 [20.35–20.67]
Time between MRI assessments (months)	-	-	24.10 [22.98–25.21]	23.99 [22.09–25.89]
Age of onset on drinking***	16.58 [16.12–17.04]	15.50 [15.06–15.94]	-	-
Average # drinks per drinking occasion***	1.81 [1.30–2.32]	7.03 [5.68–8.38]	1.84 [1.36–2.31]	8.00 [6.42–9.58]
Average # drinks per week***	1.12 [0.47–1.77]	11.00 [8.02–13.98]	1.13 [0.49–1.77]	14.89 [7.38–22.40]
Number of BD episodes^a^ (last 180 days)***	0.61 [0.03–1.19]	20.31 [14.90–25.72]	0.71 [0.17–1.26]	27.00 [17.15–36.85]
Total AUDIT score^b^ ***	1.71 [0.93–2.50]	9.60 [7.67–11.53]	1.68 [1–2.35]	9.44 [7.14–11.73]

***p ≤ 0.001.

*^a^BD episode: consumption of ≥ 50 g (females) or ≥ 70 g (males) of alcohol in one drinking occasion, raising blood alcohol concentration above 0.08 g/dL.*

*^b^In the BD group, missing scores for the second (participant 1) and the sixth and seventh items (participant 2) of the AUDIT at baseline were replaced by the BD group mean in each specific item.*

### Image Acquisition

Structural images were collected at both baseline and follow-up MRI scans on a 3T Achieva Philips body scanner (Philips Medical Systems, Best, NL) equipped with a 32-channel SENSE head coil (located at the University Hospital Complex of Santiago de Compostela, Spain). Three-dimensional T1-weighted anatomical images were acquired using a 3D turbo field-echo sequence with the following parameters: TR/TE = 7.7/3.4 ms, flip angle = 8°, FOV = 240 mm, voxel size = 0.8 mm^3^, 200 transverse slices, acquisition time = 7 min. All images were inspected to assess any artifacts or abnormal structural features.

### Image Preprocessing

#### Cross-Sectional and Longitudinal Processing Pipeline

The cortical surfaces and subcortical volumes were reconstructed and segmented using the FreeSurfer 6.0 image analysis suite^1^ (50, 51). First, preprocessing from baseline and follow-up was conducted separately using the standard cross-sectional stream. Briefly, processing included removal of non-brain tissue, automated Talairach transformation, segmentation, intensity normalization, tessellation of the gray/white matter boundary, topology correction, and surface deformation. Then, the FreeSurfer longitudinal stream^2^ was applied; this method included the creation of a within-subject template space and image from two cross-sectional time-points (baseline and follow-up) using robust registration (52, 53). Subsequent steps were initialized with common information from the within-subject template, increasing reliability and statistical power (53). For both cross-sectional and longitudinal processing pipelines, anatomical gray matter parcellations were labeled with reference to the Desikan-Killiany Atlas (54), while the subcortical segmentation was derived from Fischl et al. (55). When all reconstructions were completed, the resultant surfaces were used to calculate thickness, surface area, and cortical volume vertex-wise representations. The cortical volume is determined as the product of thickness and surface area for each cortical region; the thickness is defined as the shortest distance between the gray-white matter and pial surfaces, whereas surface area was calculated as the sum of the area within a given region on the white surface (56–58). Prior to the statistical analyses, individual thickness, surface area, and cortical volume maps were spatially smoothed using a Gaussian filter with full-width at half-maximum (FWHM) of 15 mm. Data outputs from each stage were inspected to ensure accuracy using the protocol developed by the ENIGMA consortium.^3^

In addition, based on our recent systematic review regarding the brain correlates that may be specific to BD in adolescents and young adults (5), the following bilateral regions of interest (ROIs) were selected: anterior cingulate (rostral and caudal divisions) and middle frontal gyrus (rostral and caudal divisions) defined according to the Desikan-Killiany atlas (54), and nucleus accumbens (NAcc) on the basis of the Aseg atlas (55). Consequently, cortical and subcortical structural values calculated in Freesurfer for each ROI (i.e., mean thickness in mm, surface area in mm^2^, and volume in mm^3^) were retrieved from the statistics files and exported to SPSS for statistical analysis.

### Statistical Analysis

#### Demographic and User Characteristics

Demographic and consumption variables were compared between the groups (BDs vs. controls) using Student’s *t*-tests or chi-squared tests when appropriate. For longitudinal data, mixed-models repeated-measures analyses of variance (ANOVAs) were carried out for each consumption variable with the factor time (baseline vs. follow-up) as within-subject factor and group and sex as between-subject factors. *Post-hoc* comparisons were performed using the Bonferroni adjustment for multiple comparisons. All analyses were done with SPSS (version 22).

#### Whole-Brain Surface Analyses

All group whole-brain comparisons were executed in FreeSurfer using vertex-wise general linear models (GLMs) for each hemisphere independently. The estimated total intracranial volume was included as a covariate in the analysis of surface area and cortical volume to correct volumetric data for inter-individual differences in brain sizes (59). The longitudinal change was computed using the symmetric percentage change (SPC) rate. SPC is defined as a single metric that represents the percent change with respect to the average of thickness/volume/surface area across both time-points at each vertex for each participant (53). SPC is calculated using the formula:


$$\text{SPC}=100*\frac{\mathrm{measure}\,\mathrm{at}\,\mathrm{time}\,2-\mathrm{measure}\,\mathrm{at}\,\mathrm{time}\,1}{\begin{array}{c} \left(\mathrm{interval}\mathrm{between}\mathrm{assessments}\right)*0.5*\\ \left(\mathrm{measure}\,\mathrm{at}\,\mathrm{time}\,1+\mathrm{measure}\,\mathrm{at}\,\mathrm{time}\,2\right) \end{array}}=100*\frac{\text{Rate}}{\text{Average}}$$

Both cross-sectional and longitudinal designs were conducted accounting for the interaction effects between group and sex. Whole-brain results from each GLM analysis were corrected for multiple comparisons using Monte Carlo Z simulation implemented in FreeSurfer (60), with a cluster-forming threshold of –log10 *p* = 3.3 (corresponding to *p* < 0.0005) and a cluster-wise p (CWP) threshold of < 0.05 (10.000 permutations).

#### Region of Interest Analyses

Group differences in cortical and subcortical ROI measures were tested using ANOVAs or Analyses of Covariance (ANCOVAs). The dependent variables were the mean ROI values (including SPC data) for each calculated metrics (i.e., thickness, surface area and volume [cortical and subcortical]) while group and sex were included as fixed factors. The estimated total intracranial volume was added as a covariate when outcome measures were surface area and volume (cortical and subcortical). To counteract the multiple comparison problem, the Bonferroni procedure was applied (dividing the significance level by the number of ROIs examined). Therefore, only *p*-values of < 0.00625 (α = 0.05/8) for cortical regions (i.e., anterior cingulate, middle frontal gyrus) and *p* < 0.025 (α = 0.05/2) for subcortical regions (i.e., NAcc) were considered significant. Bonferroni adjustment (*p* < 0.05) was applied to *post-hoc* comparisons where appropriate.

#### Correlation Analyses

Spearman’s rho (controlling for estimated total intracranial volume in the case of volume and surface area) were performed between morphology parameters extracted from each cluster or/and ROIs presenting significant between-group differences and the following consumption variables at follow-up: (i) average drinks per drinking occasion; (ii) total AUDIT score. All tests were performed with a statistical significance threshold set at *p* < 0.05.

## Results

### Sample Characteristics

Sample characteristics of each group for both baseline and follow-up are detailed in Table 2. No differences were observed between the groups in terms of sex, age, or interval between MRI assessments. As expected, alcohol consumption variables were significantly different between groups in both assessments: age of onset of alcohol consumption, *t*_(33)_ = 3.540, *p* = 0.001; average drinks per drinking occasion [*F*_(1,__40)_ = 111.679, *p* < 0.001, η*_*p*_*^2^ = 0.736]; average drinks per week [*F*_(1,__40)_ = 62.161, *p* < 0.001, η*_*p*_*^2^ = 0.608]; number of BD episodes [*F*_(1,__40)_ = 120.270, *p* < 0.001, η*_*p*_*^2^ = 0.750]; AUDIT score [*F*_(1,__40)_ = 93.903, *p* < 0.001, η*_*p*_*^2^ = 0.701]. Significant interactions were observed between time by sex [*F*_(1, 40)_ = 4.732, *p* = 0.036, η*_*p*_*^2^ = 0.106] and group by time by sex for AUDIT score [*F*_(1, 40)_ = 8.757, *p* = 0.005, η*_*p*_*^2^ = 0.180]. Specifically, the first interaction could be explained by a decrease in AUDIT score in females relative to males at follow-up (4.62 [95% CI = 3.33–5.90] vs. 6.47 [95% CI = 5.22–7.73]; *p* = 0.044). The second interaction revealed a decrease in AUDIT score in baseline vs. follow-up in BD females (9.79 [95% CI = 7.85–11.73] vs. 8.00 [95% CI = 5.97–10.03]; *p* = 0.016) but an increase in AUDIT score in BD males (9.41 [95% CI = 7.47–11.35] vs. 10.88 [95% CI = 8.85–12.90]; *p* = 0.047). Furthermore, BD males scored higher than BD females only at follow-up (*p* = 0.049).

### Magnetic Resonance Imaging Results

#### Cross-Sectional Analysis (Baseline and Follow-Up)

##### Baseline

Neither the whole-brain analysis, nor the ROI analysis revealed significant group differences in thickness, surface area or volume (cortical and subcortical) between BDs and controls.

##### Follow-Up

**FIGURE 2:**
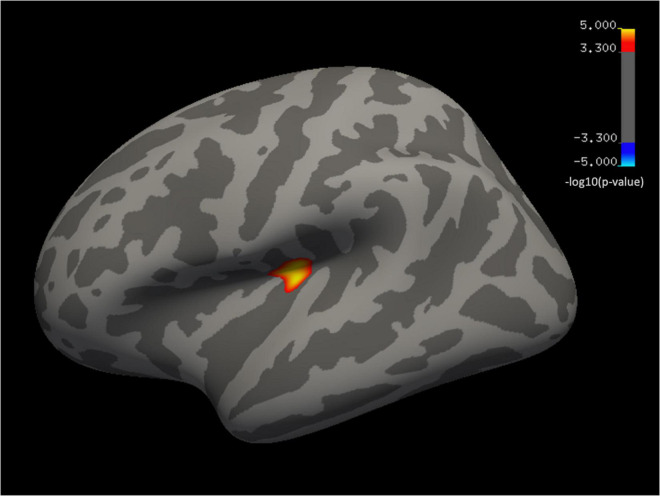
Whole-brain vertex-wise analysis. Inflated cortical convolution maps showing group differences in a cluster located in the left insula, corrected for multiple comparisons (cluster-forming threshold *p* < 0.0005 and cluster-wise p-threshold < 0.05). Significance levels are on a -log(p) scale; positive values reflect the larger surface area in BDs vs. controls (warm colors).

**FIGURE 3:**
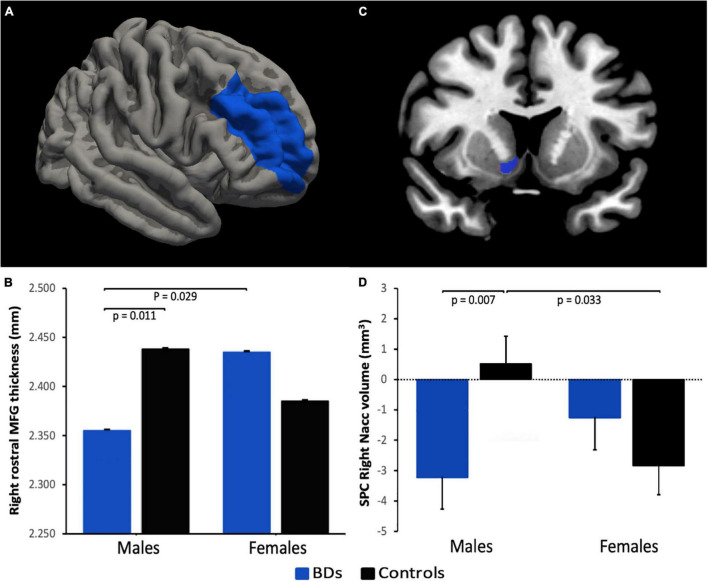
Region of interest (ROI) analysis. **(A,C)** Areas in blue illustrate the brain labels (derived from FreeSurfer atlases) used to define ROIs. **(B,D)** Bar graphs represent significant group by sex interactions in rostral middle frontal gyrus (MFG) thickness at follow-up and right nucleus accumbens (NAcc) volume across time (i.e., symmetrized percent change, SPC), respectively. Errors bars represent the standard error of the mean. NAcc image is displayed in radiological convention (i.e., left is right).

Whole-brain analysis showed that BD group, in comparison to controls, had significantly larger surface area (CWP = 0.045) in one cluster located in the left insula (MNI coordinates = –31.4, –26.2, 8.7, cluster size = 280.74 mm^2^) (Figure 2). ROI analyses on thickness revealed a group by sex interaction [*F*_(1,__40)_ = 8.857, *p* = 0.005, η*_*p*_*^2^ = 0.181] in the rostral division of the right middle frontal gyrus (Figures 3A,B), with lower thickness observed in BD males compared to controls males (*p* = 0.011). In the BD group, a reduced thickness was also observed in this region in males relative to females (*p* = 0.029).

#### Longitudinal Analysis

Whole-brain analyses found no differences in the SPC in any of the measures examined (i.e., thickness, surface area, and cortical volume). ROI analyses on subcortical volume revealed a group by sex interaction [*F*_(1,__39)_ = 7.596, *p* = 0.009, η*_*p*_*^2^ = 0.163] in the right NAcc (Figures 3C,D), showing that BD males had reduced volume over time relative to control males (*p* = 0.007); in the control group, an increase of volume in males compared to females was observed (*p* = 0.033). Regardless of alcohol consumption, a significant main effect of sex was observed in the left rostral middle frontal gyrus thickness [males < females; *F*_(1,__40)_ = 9.584, *p* = 0.004, η*_*p*_*^2^ = 0.193].

#### Relationship Between Gray Matter Structural Measures and Alcohol Consumption Variables

Whole-brain cluster in the left insula (surface area, both groups combined): the surface area was positively correlated with the average drinks per drinking occasion (rho = 0.684, *p* < 0.001) and with AUDIT score (rho = 0.682, *p* < 0.001).

Right rostral middle frontal gyrus (thickness, males): thickness of this ROI was negatively correlated with the average drinks per drinking occasion (rho = –0.593, *p* = 0.003) and with AUDIT score (rho = –0.554, *p* = 0.006).

Right NAcc (SPC volume, males): correlation analysis of this ROI showed a negative association between SPC volume and the average drinks per drinking occasion (rho = –0.542, *p* = 0.009).

## Discussion

This study aimed to examine cross-sectionally and longitudinally the effect of the BD pattern on young brain development on three different structural metrics: thickness, surface area, and volume. To this end, we tested for differences between emerging adult BDs and age-matched controls in both baseline and follow-up time-points, as well as in the SPC measure over 2 years.

### Cross-Sectional Findings (Baseline and Follow-Up)

First, contrary to our hypothesis, we did not find differences in baseline time between groups, regardless of the type of analysis employed (e.g., whole-brain analysis or ROI). These results contrast with several cross-sectional studies which have consistently associated a BD pattern in adolescence and young adulthood with structural abnormalities, especially in prefrontal regions (i.e., middle frontal gyrus, anterior cingulate), and subcortical limbic areas (ventral striatum/accumbens) (19–24). Considering the similarity between our study and those previously mentioned in relation to sample characteristics together with the consumption pattern, we believe that the conservative significance threshold used in our analysis, both in ROI-based and surface vertex approach, could help to explain this discrepancy. Nevertheless, other additional factors, such as a longer maintenance of the BD pattern before the baseline assessment (e.g., 19) or the variability in the age range of the samples (which could correspond with different neurodevelopmental stages) (e.g., 23, 24) may also contribute to the inconsistency in results (see 5 for a systematic review on structural findings and methodological details of cross-sectional BD studies).

Second, as expected and partly in agreement with our predictions, anomalies not initially observed emerged at follow-up, as well as a significant group by sex interaction. Specifically, whole-brain analyses showed that young BDs had a larger surface area in a cluster in the left insula compared to controls. The insular cortex subserves multiple brain processes, such as cognitive control, detecting interoceptive cues and emotional regulation (61). Furthermore, this brain area has been proposed as particularly relevant in the addiction cycle, mainly due to its role in the conscious urges to use drugs [for review see (62)]. Structural gray matter abnormalities implicating the insula in alcohol (63–68) and other drugs addictions (69) have been described. Interestingly, in accordance with our results, a recent study observed significantly higher bilateral insular surface in adult individuals with alcohol use disorder (68); nevertheless, it should be noted that meta-analyses of voxel-based morphometry studies in this population revealed reduced volume in the insula (70, 71). Previous longitudinal research has also shown insular structural alterations in both moderate and heavy alcohol use during emerging adulthood (30, 35, 72). However, these studies reported reductions in surface area (72) and volume (30, 35), without a consistent lateralization pattern of structural changes throughout the studies. To date, no insular anomalies have been detected in BDs, in contrast to several functional imaging studies that have shown altered brain activity (i.e., hyperactivation) in this region during decision-making (73), response inhibition (41, 74), and alcohol cue-reactivity tasks (75). We thus observed, for the first time, a larger surface area in the left insula in youth with a BD pattern. Although the surface area has not been explored as much as thickness or volume, neurodevelopment studies generally showed a decline in this feature across adolescence (76). As anticipated in the introduction, there are different possible explanations for understanding the meaning of the direction of gray matter anomalies. Our result fits with the hypothesis proposed by Squeglia et al. (25), suggesting that increases in gray matter measures in BDs may represent an alteration of healthy synaptic pruning processes due to alcohol exposure. This interpretation of a possible interference of neuromaturational processes would also be supported by other studies using BD samples with a similar university-age range, which revealed an increase in gray matter volume in cortical regions (19, 20).

On the other hand, ROI analyses reported a significant group by sex interaction on thickness in right rostral middle frontal gyrus at follow-up, showing that BD males had thinner cortices than both control males and BD females. The middle frontal gyrus is involved in executive functions, such as inhibitory control, working memory and cognitive flexibility (77). Studies focusing on young BDs have reported structural abnormalities in the middle frontal gyrus volume (19, 20, 23). Functional anomalies in this region during inhibition tasks prospectively predicted both binge and heavy drinking consumption throughout adolescence and alcohol-related problems in the future (78). Our finding is congruent with prior studies that reported reduced thickness or volume in BD males compared to control males in prefrontal regions (25, 26). Though, we found no differences in gray matter between BD females and their counterparts controls. One factor that may help explain this discrepancy is the significantly lower AUDIT score (as a measure of drinking severity) in BD females than BD males at follow-up.

### Longitudinal Findings

Another main objective of this study was to explore potential longitudinal changes (i.e., SPC) in gray matter features in youth with a continuous BD pattern. ROI analyses reported a significant group by sex interaction in the right NAcc, showing that BD males had a greater volume decline over a 2-year follow-up than control males. The NAcc has been implicated in the reinforcing properties of acute alcohol consumption and plays a crucial role in neural models of addiction (79). Previous cross-sectional studies have found structural abnormalities in NAcc volumes in university-aged BDs (22, 24). Likewise, the reduction of NAcc volumes in BDs over time observed in our study agrees with several longitudinal investigations that have associated both binge (80) and heavy alcohol use (27, 30–34) during adolescence and emerging adulthood with a decrease of cortical and subcortical gray matter volumes. Various of these studies suggest that the decline of gray matter could be interpreted as an accelerated but non-beneficial pruning due to the neurotoxic effects of alcohol. Of note, a recent mega-analysis in the framework of the IMAGE project reported significantly lower NAcc volumes in alcohol-dependent individuals relative to non-dependent controls (81). Importantly, our finding is partially in line with the cross-sectional study by Kvamme et al. (26) which found a sex-specific decrease in NAcc volumes in university-aged BD males relative to control males, whereas females showed the opposite pattern. Given the longitudinal nature of the analyses used here, our results would further indicate that the maintenance of a BD pattern is associated with sex-related abnormalities in this structure over time, linked to the number of drinks per drinking occasion. These sex-differentiated effects of alcohol during early adulthood may be due to multiple factors. For example, one reason that may partially explain the sex differences observed in our sample is the increase in drinking severity over time (follow-up vs. baseline) in BD males along with the decrease observed in BD females. It is, therefore, possible that higher alcohol consumption over time aberrantly increases pruning in BD males relative to females. Another aspect to consider are the structural differences between males and females observed in neurodevelopmental studies which have typically been interpreted in terms of the rate and timing of synaptic pruning (82, 83).

### Strengths and Limitations

To our knowledge, this is the first study on BD that combined cross-sectional data collected at two-time points over time with longitudinal change data, several morphological measures characterizing the cortical mantle (thickness, surface area, and volume), and a rigorous control for potential confounders to elucidate the structural changes in the young brain related to the BD pattern. However, despite its many strengths, our study also had some limitations to consider. Firstly, the strict exclusion criteria, together with the recruitment of subjects limited to students who completed all neuroimaging assessments and maintained the drinking pattern for a period of at least 2 years, resulted in a relatively small sample size. Secondly, the characteristics of the selected participants (university students), such as high cognitive functioning and absence of associated pathologies, might limit the representativeness of our results to other populations. Finally, future studies with more than two follow-up assessments should be conducted to explore whether the abandonment of the BD pattern would imply a reversal or attenuation of the abnormalities previously observed.

## Conclusion

Overall, our cross-sectional and longitudinal results suggest that continued BD in emerging adults may lead to structural gray matter anomalies in several regions strongly associated with reward processing, emotional regulation and executive functions. Remarkably, some abnormalities may vary depending on sex, reflecting a differential consequence of BD pattern on neuromaturation trajectories between males and females.

## Data Availability Statement

The raw data supporting the conclusions of this article will be made available by the authors, without undue reservation.

## Ethics Statement

The studies involving human participants were reviewed and approved by the Bioethics Committee of the Universidade de Santiago de Compostela. The patients/participants provided their written informed consent to participate in this study.

## Author Contributions

FC obtained funding for the study. FC, SR, MC, and SD designed the study. MC and SR were responsible for sample selection. JP-G, SS-S, and JB-R collected the data. JP-G, EC-R, and SD analyzed and interpreted the data. JP-G wrote the manuscript. All authors reviewed the manuscript and approved the submitted version.

## Conflict of Interest

The authors declare that the research was conducted in the absence of any commercial or financial relationships that could be construed as a potential conflict of interest.

## Publisher’s Note

All claims expressed in this article are solely those of the authors and do not necessarily represent those of their affiliated organizations, or those of the publisher, the editors and the reviewers. Any product that may be evaluated in this article, or claim that may be made by its manufacturer, is not guaranteed or endorsed by the publisher.

## Footnotes

1
https://surfer.nmr.mgh.harvard.edu/

2
https://surfer.nmr.mgh.harvard.edu/fswiki/LongitudinalTwoStageModel

3
http://enigma.ini.usc.edu/protocols/imaging-~protocols/
